# Influenza immunization in Canada’s low-income population

**DOI:** 10.1186/1471-2458-14-740

**Published:** 2014-07-21

**Authors:** Jennifer Leigh Hobbs, Jane A Buxton

**Affiliations:** 1 School of Population and Public Health, University of British Columbia, Vancouver, BC, Canada

**Keywords:** Immunization, Vaccination, Influenza, Flu, Socioeconomic status, Low-income, Social determinants, Health, Canada

## Abstract

**Background:**

Immunization offers the best protection from influenza infection. Little evidence describes disparities in immunization uptake among low-income individuals. Higher rates of chronic disease put this population at increased risk of influenza-related complications. This analysis examines if the type of main source of household income in low-income groups affects influenza immunization uptake. We hypothesized that individuals on social assistance have less access to immunization compared to those with employment earnings or seniors’ benefits.

**Methods:**

Data was obtained from the Canadian Community Health Survey annual component 2009-2010. A total of 10,373 low-income respondents (<20,000$ Canadian per annum) were included. Logistic regression, stratified according to type of provincial publicly funded immunization program, was used to examine the association between influenza immunization (in the last 12 months) and main source of household income (employment earnings; social assistance as a combination of employment insurance or worker’s compensation or welfare; or seniors’ benefits).

**Results:**

Overall, 32.5% of respondents reported receiving influenza immunization. In multivariable analysis of universal publicly funded influenza immunization programs, those reporting social assistance (AOR 1.24, 95% CI 1.02-1.51) or seniors’ benefits (AOR 1.56, 95% CI 1.23-1.98) were more likely to be immunized compared to those reporting employment earnings. Similar results were observed for high-risk programs.

**Conclusions:**

Among the low-income sample, overall influenza immunization coverage is low. Those receiving social assistance or seniors’ benefits may have been targeted due to higher rates of chronic disease. Programs reaching the workforce may be important to attain broader coverage. However, CCHS data was collected during the H1N1 pandemic influenza, thus results may not be generalizable to influenza immunization in non-pandemic years.

## Background

Influenza infection poses a significant public health burden affecting millions of Canadians each year resulting in 20,000 hospitalizations and 4,000 to 8,000 deaths, particularly among high risk groups [1,2]. Influenza immunization offers the best protection from infection and disease and at the national level is recommended for all Canadians over six months of age [1]. Delivery of influenza immunization programs occurs at the provincial level. Some publicly funded provincial programs are universal in scope and offer the vaccine free of charge to all residents, while others are limited to high risk groups and offer the vaccine free of charge only to individuals at high risk of complications from infection and/or individuals providing care for those at high risk [3]. Further details describing high risk groups are provided by the National Advisory Committee on Immunization statement on seasonal influenza immunization [4]. In 2007 to 2008, national influenza immunization rates reached 31% among Canadians 12 years of age and older [5].

However, immunization coverage is not equal among all segments of the Canadian population. The influence of socio-economic and demographic factors such as age, gender, chronic disease status, ethnicity, access to health care, education and income on influenza immunization have been well described [6-11]. In a recent study, older age, having a chronic disease, and having a medical doctor were associated with influenza immunization uptake [11]. However, in Ontario, unlike other provinces, no relationship between immunization and age and income was reported suggesting more equitable access to vaccine perhaps due to the universal scope of the publicly funded immunization program [11].

While factors influencing influenza immunization in certain sub-populations have been examined, little evidence exists describing disparities among low-income individuals [12]. Higher rates of chronic disease put this population at increased risk of influenza-related complications [12,13]. Access to immunization programs may not be uniform and barriers may be distinct from those described for the general population and other sub-populations. Two studies in the United States assessing rates of influenza immunization in disadvantaged urban areas found that access to social services, health services or health insurance were important determinants [12,13]. However, findings likely need to be interpreted with caution in the Canadian context due to differences in immunization program delivery within public and private health care systems [8].

This analysis aims to examine the influence of the source of household income (employment, social assistance, or seniors’ benefits) on influenza immunization among low-income individuals, defined as individuals reporting a total annual household income of less than $20,000, in the Canadian population and whether this differs according to the type of publicly funded program (universal or high risk) available in the province. Source of income may serve as a surrogate measure to identify potentially marginalized individuals within the low-income group. Awareness of influenza immunization programs may be through the workplace, seniors’ residence immunization clinics, and more frequent physician visits with age. Therefore, individuals on social assistance may have less access to and awareness of immunization programs compared to those with employment earning or on seniors’ benefits.

## Methods

### Study design

Data was obtained from the Public Use Microdata File of the Canadian Community Health Survey (CCHS) annual component January 2009 to December 2010. The CCHS is a cross-sectional survey that collects information on the health status, health care utilization, and health determinants of the Canadian population. Survey data was collected using a multistage stratified cluster design from a representative sample of 124,188 individuals aged 12 or older in all 10 provinces and 3 territories. The sample is representative of approximately 98% of the Canadian population. The 2% not represented includes individuals living on Indian Reserves, Crown Lands, those residing in institutions, full-time members of the Canadian Forces, and residents of certain remote regions. Further details pertaining to the CCHS sampling methodology are reported elsewhere [14].

### Study sample

**Figure 1:**
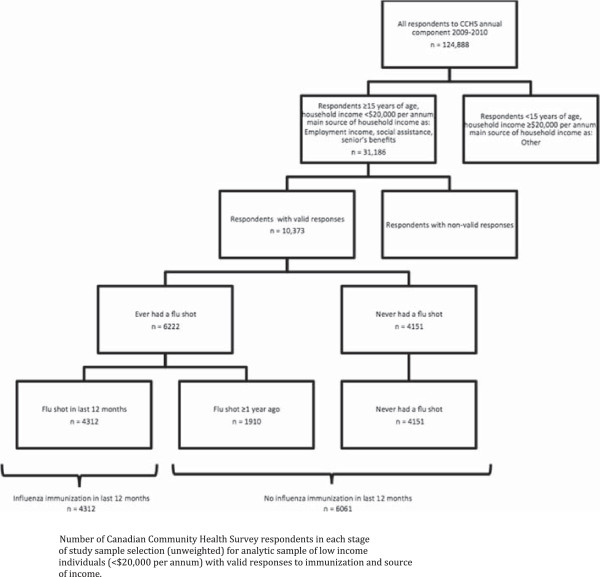
Number of Canadian Community Health Survey respondents in each stage of study sample selection.

This analysis was restricted to individuals 15 years of age or older reporting a total annual household income of less than $20,000, consistent with the low income cut off estimated by Statistics Canada (Figure 1) [15]. Individuals not stating annual household income were not eligible for the study sample. Individuals reporting “other” (dividends, interest, child support, alimony, other, or no income) as the main source of household income were excluded based on conceptual considerations. Non-valid responses, including individuals who did not state their main source of household income, influenza immunization status, or a response for confounding variables, were also excluded.

### Study variables

The outcome variable, receiving influenza immunization in the last 12 months, was constructed from the CCHS questions “Have you ever had a seasonal flu shot?” and “When did you have your last seasonal flu shot?”. A comparison was made between individuals who reported influenza immunization in the last 12 months and those who did not report influenza immunization in the last 12 months. Individuals who did not report influenza immunization in the last 12 months included those immunized one to two years ago, two or more years ago, and those reporting never receiving an influenza immunization (Figure 1). The primary explanatory variable, main source of household income in the last 12 months, was listed in the CCHS as three categories: employment earnings; social assistance as a combination of employment insurance or worker’s compensation or welfare; or seniors’ benefits. Inclusion of confounding variables (age, gender, education, immigration, and self-perceived health) in the adjusted multivariable regression analysis was based on previous study findings and conceptual considerations [6-12]. Additional variables (not included in the multivariable regression analysis) used to describe the sample included chronic disease (as a combination of asthma, bronchitis, emphysema, chronic obstructive pulmonary disease, diabetes, cancer, and heart disease), occupation group, and reasons for non-immunization (no flu shot). Reasons for non-immunization were captured in the CCHS as a number of questions, including “Did not think it was necessary” and “Have not gotten around to it”.

### Analysis

Univariable analysis used logistic regression to determine unadjusted odds ratios and 95% confidence intervals measuring the association between influenza immunization and the primary explanatory variable, source of household income, and potential confounders. Confounding variables showing an association in the direction expected, based on a priori knowledge and conceptual considerations, were included in the multivariable model. A large proportion of individuals over 65 years of age reported seniors’ benefits as the main source of household income, thus this relationship was further explored to ensure assumptions required for statistical tests were met. The multivariable model was developed with manual step-wise entry of each confounder. Analysis was stratified based on provincial influenza immunization program type. Provinces and territories with publicly funded universal programs (defined as provinces offering the vaccine free of charge to all residents), those with publicly funded high risk programs (defined as provinces offering the vaccine free of charge to individuals at high risk for complications from infection and/or individuals providing care for those at high risk), and those that implemented publicly funded universal programs during the CCHS data collection period (referred to as recent universal programs) were considered separately.

Sensitivity analyses compared individuals reporting influenza immunization in the last 12 months and those who had never received an influenza immunization to investigate whether or not the group reporting never receiving an influenza immunization was unique. Further sensitivity analysis explored the relationship between influenza immunization and source of household income with a sample restricted to individuals of low-income and low education (excluded post-secondary graduates). Additional variables (not included in the multivariable regression analysis) were used to provide descriptive results (proportions) where appropriate.

Power was sufficient overall and for each stratum (at least 0.92, at an alpha of 0.05), with the exception of the stratum including provinces with universal programs (where power was 0.54). Therefore, results from this stratum should be interpreted with caution. In order to produce estimates representative of the Canadian population, probability sampling weights were constructed for the analytic sample from Statistics Canada survey weights. The constructed probability weights were applied in all analyses to account for the sampling methodologies used in the CCHS. All analyses were done using SAS 9.3 for Windows (SAS Institute).

## Results

### Study sample

A large number of respondents (20,524 or 18.3% of those over 15) were excluded from the sample since annual household income was not stated. Influenza immunization coverage was similar among the income not stated and income under $20,000 categories (OR 0.92, 95% CI 0.88-0.98). However, source of income differed among the income not stated and income under $20,000 categories. Individuals with an income under $20,000 were more likely to report social assistance as the main source of household income. Among individuals 15 years of age or older who reported an annual household income under $20,000, an additional 951 (11.9%) reporting “other” source of income were excluded. Influenza immunization was similar among respondents in the “other” category. An additional 922 respondents were excluded due to non-valid responses for influenza immunization status (203), main source of household income (352) and confounders; education (285), immigrant status (62), and self-perceived health (20). Respondents excluded due to missing data did not differ significantly on influenza immunization status. The final sample size consisted of 10,373 respondents, representing 8.4% of the original CCHS sample (Figure 1).

### Baseline characteristics

A large proportion of older individuals were represented in the final analytic sample. The median age of respondents was the 50 to 54 year age category, representing 7.5% of the sample (Table 1). More females (59.3%) and non-immigrants (74.7%) were included. Almost half (45.7%) reported post-secondary graduation; however, 30.4% also reported not completing secondary school. Few respondents (8.9%) reported poor self-perceived health.

**Table 1:** Characteristics of low-income sample, relationship between household income and confounders, and influenza immunization

	**Overall study sample**		**Influenza immunization (in last 12 months)**	
	**Unweighted n (n = 10373)**	**%**	**Yes (%)**	**No (%)**
**Type of publicly funded influenza immunization program**				
Universal^*^	3279	34.9	40.2	32.3
High risk^†^	4841	49.5	41.9	53.2
Recent universal^‡^	2253	15.6	17.9	14.5
**Influenza immunization (in last 12 months)**				
Yes	4312	32.5		
No	6061	67.5		
Over 12 months ago	1910	20.3		
Never	4151	47.2		
**Main source of household income**				
Employment income	2189	34.7	18.4	42.6
Social assistance^§^	2516	29.2	23.5	31.9
Seniors’ benefits	5668	36.1	58.2	25.5
**Age (5 year category)**				
Median (50-54)	733	7.5	6.5	8.0
**Gender**				
Male	3565	40.7	31.4	45.1
Female	6808	59.3	68.6	54.9
**Immigrant**				
Yes	1419	25.2	25.3	25.2
No	8954	74.7	74.7	74.8
**Highest level of household education**				
< Secondary	4235	30.4	39.1	26.1
Secondary graduate	1589	15.0	15.3	14.9
Some post-secondary	753	9.0	6.8	10.0
Post-secondary graduate	3796	45.7	38.8	49.0
**Self-perceived health**				
Poor	1073	8.9	11.6	7.6
Fair	2355	19.4	27.1	15.7
Good	3374	31.7	32.3	31.4
Very good	2492	25.0	21.2	26.9
Excellent	1079	15.0	7.9	18.5

^*^Provinces and territories with universal publicly funded programs included; Ontario, Nunavut, the Northwest Territories and the Yukon.

^†^Provinces with publicly funded high risk programs included; Newfoundland and Labrador, New Brunswick, Quebec, British Columbia, and Prince Edward Island (Prince Edward Island provides the influenza vaccine free of charge but charges a fee for vaccine administration for non high-risk groups).

^‡^Provinces that implemented a universal publicly funded program during the study period included; Nova Scotia, Alberta, Saskatchewan, and Manitoba.

^§^Employment insurance or worker’s compensation or welfare.

Characteristics of low-income (<$20,000 per annum) Canadian Community Health Survey sample 2009-2010, limited to valid responses (weighted analysis).

### Outcome and exposure

Among the sample, 32.5% reported receiving an influenza immunization within the last 12 months. Of those not reporting an influenza immunization in the last 12 months, 47% reported never receiving an influenza immunization. The main source of household income was fairly evenly distributed in the sample, with 34.7% of respondents reporting employment income as their main source of household income, 29.2% reporting social assistance, and 36.1% reporting seniors’ benefits. Among those reporting influenza immunization in the last 12 months, the majority (58.2%) reported seniors’ benefits, while only 18.4% reported employment income.

### Analysis

In the univariable models, stratified according to type of publicly funded influenza immunization program, the odds of influenza immunization was higher among individuals reporting social assistance (defined as employment insurance or worker’s compensation or welfare) and significantly higher among individuals reporting seniors benefits compared to those reporting employment income (Table 2). In the multivariable models, stratified by type of immunization program and adjusted for age, gender, immigration, education, and self-perceived health; the odds of influenza immunization and the variability of the estimates were reduced but remained elevated for both individuals reporting social assistance and seniors benefits compared to those reporting employment earnings regardless of the type of provincial publicly funded program (Table 2). Adjusting for chronic disease did not alter the findings, thus was not included as a confounder in the final model (results not shown).

**Table 2:** Odds of influenza immunization for main source of household income by influenza immunization program type

	**Universal program**		**Recent universal program**		**High risk program**	
	**Unadjusted OR (95% CI)**	**Adjusted**^**§ **^**OR (95% CI)**	**Unadjusted OR (95% CI)**	**Adjusted**^**§ **^**OR (95% CI)**	**Unadjusted OR (95% CI)**	**Adjusted**^**§ **^**OR (95% CI)**
**Source of income**						
Employment income	Reference	Reference	Reference	Reference	Reference	Reference
Social assistance^*^	1.26 (1.06-1.51)	1.24 (1.02-1.51)	1.67 (1.24-2.24)	1.27 (0.91-1.76)	2.54 (2.08-3.11)	1.49 (1.20-1.86)
Seniors’ benefits	3.95 (3.33-4.70)	1.56 (1.23-1.98)	4.72 (3.69-6.04)	1.90 (1.29-2.79)	8.59 (7.16-10.31)	2.09 (1.62-2.70)
Age (5 year increments^†^)	1.21 (1.19-1.24)	1.16 (1.13-1.19)	1.20 (1.17-1.23)	1.12 (1.08-1.18)	1.32 (1.30-1.35)	1.21 (1.18-1.25)
**Gender**						
Male	Reference	Reference	Reference	Reference	Reference	Reference
Female	1.97 (1.71-2.28)	1.65 (1.42-1.93)	2.18 (1.76-2.70)	1.97 (1.56-2.48)	1.53 (1.35-1.74)	1.07 (0.93-1.24)
Immigrant						
Yes	Reference	Reference	Reference	Reference	Reference	Reference
No	0.94 (0.82-1.08)	1.07 (0.91-1.25)	1.55 (1.16-2.08)	1.24 (0.89-1.72)	1.09 (0.94-1.28)	0.80 (0.67-0.95)
**Highest level of household education**						
< Secondary	Reference	Reference	Reference	Reference	Reference	Reference
Secondary graduate	0.66 (0.54-0.78)	0.98 (0.79-1.23)	0.67 (0.48-0.94)	1.14 (0.79-1.65)	0.64 (0.53-0.78)	0.99 (0.79-1.22)
Some post-secondary	0.39 (0.30-0.51)	0.69 (0.51-0.92)	0.68 (0.47-0.96)	1.61 (1.08-2.41)	0.37 (0.28-0.49)	0.67 (0.49-0.90)
Post-secondary graduate	0.66 (0.56-0.78)	1.10 (0.91-1.34)	0.62 (0.49-0.78)	1.28 (0.98-1.68)	0.39 (0.34-0.45)	0.77 (0.66-0.91)
**Self-perceived health**						
Poor	2.52 (1.88-3.38)	1.42 (1.03-1.95)	3.45 (2.16-5.50)	1.85 (1.11-3.10)	4.45 (3.35-5.91)	2.71 (1.99-3.68)
Fair	3.33 (2.58-4.31)	2.17 (1.64-2.86)	3.60 (2.40-5.40)	2.11 (1.35-3.29)	4.67 (3.69-5.91)	2.66 (2.06-3.45)
Good	1.79 (1.40-2.29)	1.21 (0.93-1.57)	2.56 (1.74-3.79)	1.70 (1.11-2.59)	2.84 (2.28-3.55)	1.88 (1.48-2.40)
Very good	1.89 (1.47-2.43)	1.62 (1.24-2.12)	1.50 (0.99-2.26)	1.19 (0.77-1.84)	1.78 (1.40-2.26)	1.47 (1.13-1.91)
Excellent	Reference	Reference	Reference	Reference	Reference	Reference

^*^Employment insurance or worker’s compensation or welfare.

^†^Odds of influenza immunization for a 5 year increase in age (up to 80 years).

^§^Adjusted for age, gender, immigration, education, and self-perceived health.

Unadjusted and adjusted multivariable logistic regression results for odds of influenza immunization (yes in last 12 months) associated with main source of household income stratified by type of publicly funded influenza immunization program, low-income (<$20,000 per annum) Canadian Community Health Survey sample 2009-2010 (weighted analysis).

Among provinces with publicly funded universal influenza immunization programs, the odds of influenza immunization were increased for individuals reporting social assistance (AOR 1.24, 95% CI 1.02-1.51) and for individuals reporting seniors’ benefits (AOR 1.56, 95% CI 1.23-1.98) compared to individuals reporting employment income. Similar results were observed in provinces with recent universal and high-risk programs. The odds of influenza immunization was increased among provinces with recent universal programs for individuals reporting social assistance (AOR 1.27, 95% CI 0.91-1.76) and seniors’ benefits (AOR 1.90, 95% CI 1.29-2.79) and was greatest among provinces with high risk programs for individuals reporting social assistance (AOR 1.49, 95% CI 1.20-1.86) and seniors’ benefits (AOR 2.09, 95% CI 1.62-2.70) compared to individuals reporting employment income. All 95% CIs excluded ‘1’, with the exception of the CI for individuals reporting social assistance for provinces with recent universal programs. Within each stratum, all 95% CIs overlapped but did not include point estimates, with the exception of the CI for individuals reporting seniors’ benefits for provinces with universal programs.

For the confounding variables, the odds of influenza immunization increased with age and were higher among females in both univariable and multivariable models. The odds of influenza immunization were also higher for those with poor self-perceived health, with the exception of the multivariable model for high risk programs. In both univariable and multivariable models there did not appear to be a strong relationship between influenza immunization and immigration (confidence intervals included ‘1’). The relationship between influenza immunization and education was also less clear, without a consistent trend in univariable and multivariable models.

For the additional variables (not included in the multivariable regression analysis), chronic disease was higher among individuals reporting social assistance and seniors’ benefits (29.7% and 52.3% respectively) compared to those reporting employment earnings (18.0%). Investigation of reasons for non-immunization demonstrated that among those that did not report influenza immunization in the last 12 months, the most common reasons for non-immunization included “Did not think it was necessary” and “Have not gotten around to it”. Furthermore, rates of immunization were lowest among individuals reporting occupations related to trades or transport and equipment operator, and primary industry or processing or manufacturing and utilities (results not shown).

## Discussion

Among the low-income population, it was hypothesized that individuals reporting social assistance as their main source of household income would have less access to and awareness of influenza immunization programs compared to those reporting employment earnings or seniors’ benefits. This association, however, does not appear to exist. Among a national sample of low-income individuals those reporting social assistance and seniors’ benefits are more likely to report receiving influenza immunization in the last 12 months compared to those reporting employment income, regardless of the type of provincial publicly funded program. The higher odds of influenza immunization observed for those receiving social assistance or seniors’ benefits may be a result of the fact that these individuals represent high risk groups targeted by immunization programs. Rates of chronic disease were higher among those reporting social assistance and seniors’ benefits (29.7% and 52.3% respectively) compared to the healthier workforce (18.0%). Higher rates of influenza immunization uptake among individuals with chronic conditions (and over 65 years of age) are supported by the literature [6,11,16]. Furthermore, since findings were consistent for provinces with both high risk and universal programs, this suggests high risk groups are being immunized regardless of the type of provincial publicly funded program. Public health campaigns appear to be effective at reaching those most at risk of influenza-related complications, however overall coverage remains low.

Findings were consistent with a study among disadvantaged urban areas in the United States where access to social services was found to be an important determinant of influenza immunization [12]. No comparable studies among low-income individuals in the Canadian context exist. However, Canadian studies have examined the influence of socio-economic indicators on influenza immunization and have reported an increasing likelihood of immunization with greater income [11,16]. Thus, a lower overall rate of influenza immunization was expected in the study sample. However, the rate of influenza immunization in the low-income study sample was similar to that of the general population [16,5]. While this finding is not consistent with the influence of income reported in Canadian studies, a recent review stated the influence of income on influenza immunization has been inconsistent [10].

Since the low-income employed population appears to be healthier (lower rates of chronic disease) than those receiving social assistance or seniors’ benefits, coverage rates may be lowest among this population due to less perceived need for immunization, regardless of access. Investigation of reasons for non-immunization demonstrated that among those that did not report influenza immunization in the last 12 months, the most common reasons for non-immunization included a lack of perceived need for immunization (“Did not think it was necessary” and “Have not gotten around to it”) rather than access to health care services. Similar findings have been reported elsewhere [17].

Several limitations arise due to the cross-sectional and self-reported nature of the data. Despite self-reporting, confounding variables previously described as determinants of influenza immunization showed associations in the direction expected adding face validity to the main finding. Also, influenza immunization rates were similar to those estimated for Canadian population suggesting influenza immunization status was reported accurately [5,16]. However, there may have been a social desirability reporting bias where high risk individuals over-reported influenza immunization. The use of self-reported data led to further limitations in describing income earning and income source. It is likely that a number of low income individuals did not state their earnings thus were excluded from the analysis. Furthermore, low-income individuals may move above and below the low income cut off as their source of income changes. The income decile and income source reported may have differed from that at the time of influenza immunization. Furthermore, CCHS data was collected during the H1N1 pandemic influenza, thus results may not be generalizable to influenza immunization in non-pandemic years [10].

Defining populations with higher rates of chronic disease, thus increased risk of influenza-related complications, is challenging. Increased risk of influenza-related complications is likely the result of a number of socio-economic factors, including income, education, and occupation, but for the purposes of this study was limited to income. However, findings were also consistent a sensitivity analysis that further restricted the sample to individuals with low education (results not shown).

The structure of the CCHS questionnaire also led to limitations. The survey grouped individuals reporting various types of social assistance into a single category. However, immunization rates may vary with type of social assistance. Low immunization rates have been reported among populations with a high number of individuals on welfare, such as Vancouver’s Downtown East-side [18]. Due to the CCHS categorization of social assistance lower rates of immunization among individuals on welfare could have been masked by higher rates among individuals on employment insurance or worker’s compensation. Furthermore, as analysis was done with the Public Use Microdata File, probability sampling weights constructed from Statics Canada survey weights, rather than bootstrapped weights, were used. Bootstrapped weights provide a more precise measure of the variability around the estimates.

## Conclusions

When implementing influenza immunization programs it is important to identify populations with low coverage and recognize the factors that affect immunization uptake. This study was unique in investigating influenza immunization among the low-income population in Canada and demonstrated that while overall coverage among the low-income population is low, public health efforts appear to be reaching high risk individuals. However there is a need, particularly with respect to education and awareness, to improve immunization coverage among the low-income workforce. Studies have demonstrated the public health benefits of widespread influenza immunization with reduced rates of influenza-related mortality and morbidity [19]. The low-income employed population may be an important group to reach to attain broader influenza immunization coverage, thus greater protection in the community, in both provinces with universal and high risk publicly funded programs.

To further describe barriers to influenza immunization among the low-income population, future studies should investigate influenza immunization among the workforce by identifying occupation groups with the lowest coverage. This study found that rates of immunization were lowest among individuals reporting occupations related to trades or transport and equipment operator, and primary industry or processing or manufacturing and utilities. Further description of influenza immunization among occupation groups could be important in designing public health efforts. Studies should also investigate the association between influenza immunization and income source using finer social assistance categories (in particular a separate category for individuals on welfare) to determine if the association observed in this study is consistent across different types of social assistance.

## Competing interests

The authors declare that they have no competing interests.

## Authors’ contributions

JLH conceived of the study, performed the analysis, and drafted the manuscript. JAB contributed to the interpretation of the findings and manuscript revisions. All authors read and approved the final manuscript.

## Pre-publication history

The pre-publication history for this paper can be accessed here:

http://www.biomedcentral.com/1471-2458/14/740/prepub

## References

[B1] Public Health Agency Canada The flu shothttp://www.fightflu.ca/fight-combattre-eng.php

[B2] Health Canada It’s your health - influenzahttp://www.hc-sc.gc.ca/hc-ps/dc-ma/influenza-eng.php

[B3] Public Health Agency Canada Public funding for influenza vaccination by province/territoryhttp://www.phac-aspc.gc.ca/im/ptimprog-progimpt/fluvacc-eng.php

[B4] National Advisory Committee on Immunization Statement of seasonal influenza vaccine for 2013-2014 Can Communicable Dis Rep 2013 39 http://www.phac-aspc.gc.ca/publicat/ccdr-rmtc/13vol39/acs-dcc-4/index-eng.php 10.14745/ccdr.v39i00a04 PMC6802458 31701948

[B5] BC Centre for Disease Control Canadian community health survey results influenza immunization coverage http://www.bccdc.ca/NR/rdonlyres/A650CC1E-76F3-4827-91B2-3B65BECDB168/51128/CCHS20070820110601.pdf

[B6] Chen Y Yi QL Wu J Li F Chronic disease status, self-perceived health and hospital admissions are important predictors for having a flu shot in Canada Vaccine 2007 25 42 7436 7440 17825962 10.1016/j.vaccine.2007.08.003

[B7] Vozoris NT Lougheed MD Influenza vaccination among Canadians with chronic respiratory disease Respir Med 2009 103 1 50 58 18818066 10.1016/j.rmed.2008.08.004

[B8] Quach S Hamid JS Pereira JA Heidebrecht CL Deeks SL Crowcroft NS Quan SD Brien S Kwong JC Ethnic disparities in influenza vaccination in Canada Can Med Assoc J 2012 24 15 1673 1681 22966054 10.1503/cmaj.111628 PMC3478352

[B9] Bish A Yardley L Nicoll A Michie S Factors associated with uptake of vaccination against pandemic influenza: a systematic review Vaccine 2011 29 38 6472 6484 21756960 10.1016/j.vaccine.2011.06.107

[B10] Brien S Kwong JC Buckeridge DL The determinants of 2009 pandemic a/H1N1 influenza vaccination: a systematic review Vaccine 2012 30 7 1255 1264 22214889 10.1016/j.vaccine.2011.12.089

[B11] Polisena J Chen Y Manuel D The proportion of influenza vaccination in Ontario, Canada in 2007/2008 compared with other provinces Vaccine 2012 30 11 1981 1985 22245605 10.1016/j.vaccine.2012.01.009

[B12] Bryant WK Ompad DC Sisco S Blaney S Glidden K Phillips E Vlahov D Galea S Determinants of influenza vaccination in hard-to-reach urban populations Prev Med 2006 43 1 60 70 16684559 10.1016/j.ypmed.2006.03.018

[B13] Armstrong K Berlin M Schwartz JS Propert K Ubel PA Barriers to influenza immunization in a low-income urban population Am J Prev Med 2001 20 1 21 25 11137770 10.1016/s0749-3797(00)00263-4

[B14] Statistics Canada Canadian Community Health Survey Annual component 2011 http://www23.statcan.gc.ca/imdb/p2SV.pl?Function=getSurvey&SDDS=3226&lang=en&db=imdb&adm=8&dis=2

[B15] Statistics Canada Low Income Cut-Offs 2012 http://www.statcan.gc.ca/pub/75f0002m/2009002/s2-eng.htm

[B16] Kwong JC Rosella LC Johansen H Trends in influenza vaccination in Canada, 1996/1997 to 2005 Health Rep 2007 18 4 1 11 18074993

[B17] Chambers CT Buxton JA Koehoorn M Consultation with health care professionals and influenza immunization among women in contact with young children Can J Public Health 2010 101 1 15 19 20364531 10.1007/BF03405554 PMC6973855

[B18] Weatherill SA Buxton JA Daly PC Immunization programs in non-traditional settings Can J Public Health 2004 95 2 133 137 15074905 10.1007/BF03405781 PMC6975862

[B19] Kwong JC Stukel TA Lim J McGeer AJ Upshur RE Johansen H Sambell C Thompson WW Thiruchelvam D Marra F Svenson LW Manuel DG The effect of universal influenza immunization on mortality and health care use PLoS Med 2008 5 10 e211 18959473 10.1371/journal.pmed.0050211 PMC2573914

